# Two-Year Change in Serum Total Cholesterol Is Associated With Incident Ischemic Stroke: Results From the Kailuan Study

**DOI:** 10.3389/fneur.2021.710083

**Published:** 2021-09-29

**Authors:** Yu Wang, Anxin Wang, Yingting Zuo, Shouling Wu, Xingquan Zhao

**Affiliations:** ^1^China National Clinical Research Center for Neurological Diseases, Beijing, China; ^2^Department of Neurology, Beijing Tiantan Hospital, Capital Medical University, Beijing, China; ^3^Department of Epidemiology and Health Statistics, School of Public Health, Capital Medical University, Beijing, China; ^4^Beijing Municipal Key Laboratory of Clinical Epidemiology, Beijing, China; ^5^Department of Cardiology, Kailuan General Hospital, North China University of Science and Technology, Tangshan, China; ^6^Research Unit of Artificial Intelligence in Cerebrovascular Disease, Chinese Academy of Medical Sciences, Beijing, China

**Keywords:** total cholesterol, risk factor, ischemic stroke, intracerebral hemorrhage, incidence

## Abstract

**Background and Purpose:** Compared with one single measurement, dynamic change of lipid parameter calculated by repeated measurements has been recognized as a potential biometric to make stroke risk assessments. Total cholesterol (TC) is an important risk factor for stroke, but the relationship between TC change and incident stroke has not been investigated thoroughly. We thus aimed to explore the association between 2-year TC change and the risk of incident stroke, both ischemic and hemorrhagic, in the general population.

**Methods:** From June 2006 to October 2007, a total of 70,999 participants with complete TC value at baseline (2006–2007) and the second examination (2008–2009) were included in our study. The change of TC was calculated as the 2-year follow-up TC subtracting baseline TC. Cox proportional hazards regression analysis was used to evaluate the association between the tertile of TC change and risk of incident stroke and stroke subtypes.

**Results:** A total of 2,815 cases of stroke events were identified with a median follow-up period of 9.0 years. After adjusting for baseline TC and confounding factors, 2-year TC change was independently associated with increased risk of total stroke (HR 1.07, 95% CI 1.02–1.12) and ischemic stroke (HR 1.08, 95% CI 1.03–1.13) per SD (1.04 mmol/L) increase, while no significant association was obtained between TC change and intracerebral hemorrhage (*p* = 0.659).

**Conclusions:** Increased 2-year TC change is associated with an elevated risk of incident total stroke and ischemic stroke, irrespective of the baseline TC value. Maintaining a sustained ideal level of TC is important for stroke prevention.

## Introduction

China bears the heaviest stroke burden worldwide (1), with an overall stroke incidence of 345.1 per 100,000 person-years (2). Based on the nationally representative survey, the proportion of ischemic stroke (IS), intracerebral hemorrhage (ICH), and subarachnoid hemorrhage (SAH) was 69.9, 23.8, and 4.4%, respectively (2). To reduce the ensuing huge health and economic burdens, preventive strategies for stroke, either ischemic or hemorrhagic, are of great importance.

Hyperlipidemia is a well-established causal factor for atherosclerotic cerebrovascular disease. Many studies reported that high total cholesterol (TC) level at baseline was associated with an elevated risk of IS (3–8), while others failed to obtain such a relationship (9, 10). Likewise, no agreement was reached on the association between TC and hemorrhagic stroke (11, 12). Whether lower lipid levels have an increased hemorrhagic risk is worth further discussion. Of note, previous studies focused on one single measurement of TC, about which individual variation may exist due to short-term personal diet and physical activity. Recently, the National Health Screening Programs of Korea reported that increased TC change (calculated as follow-up minus baseline values) was associated with high cardiovascular risk (13). Despite limited data, the change of TC level has been recognized as a potential biometric to make risk assessments.

Therefore, we aimed to investigate the association between the 2-year change in TC level and incident stroke, with attention to stroke subtype and population subgroup, in a Chinese community-based cohort study.

## Materials and Methods

### Study Design and Population

The study was conducted in accordance with the Declaration of Helsinki and was approved by the Ethics Committees of the Kailuan General Hospital and Beijing Tiantan Hospital. All participants or their legal representatives provided written informed consent (Trial registration: ChiCTR-TNRC-11001489).

**Figure 1:**
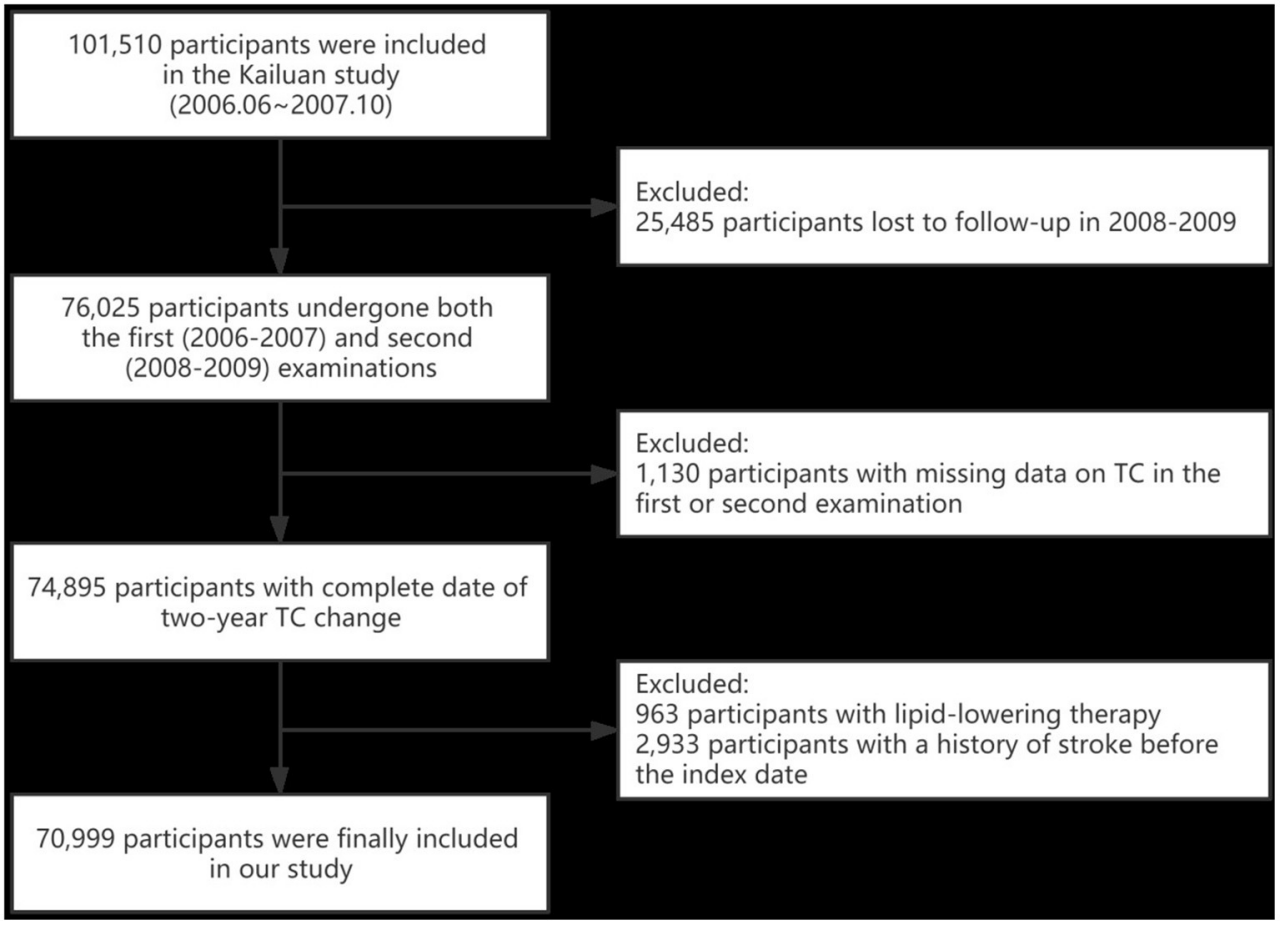
Flow chart for selection of study participants. TC, total cholesterol.

The Kailuan study is a community-based, prospective, cohort study conducted in a coal mine industry located in Tangshan City, Hebei province, China. Details about the design and methods of the study have been reported previously (14, 15). From June 2006 to October 2007, 101,510 participants were recruited at baseline and followed up biennially with questionnaire assessment, clinical examination, and laboratory test in the Kailuan study. We identified 76,025 participants who had undergone health check-ups for both the first (2006–2007) and second (2008–2009) examinations. The index date was the date after the second examination, with follow-up every 2 years until December 31, 2017. We excluded 1,130 participants with missing data on TC in the first or second examination. Moreover, 963 participants with lipid-lowering therapy, together with 2,933 participants with a history of stroke ahead of the index date were excluded. Eventually, a total of 70,999 participants were included in our study (Figure 1). Baseline characteristics between the included and excluded participants are shown in Supplementary Table 1.

### Measurement of TC and Calculation of TC Change

In each clinical examination, blood samples were obtained from the antecubital vein after an overnight fast and analyzed within 4 h of preparation using an auto-analyzer (Hitachi 747; Hitachi, Tokyo, Japan) at the central laboratory of the Kailuan hospital. TC was measured using the enzymatic end-point method. TC levels at baseline (2006–2007) and the second examination (2008–2009) were both recorded. The change of TC was calculated as the 2-year follow-up TC subtracting baseline TC. Participants were then divided into three groups according to the tertiles of TC change, and the lowest tertile was defined as the reference group.

### Measurement of Potential Covariates at Baseline

Demographic and clinical characteristics including age, sex, smoking status, drinking status, physical activity, and past medical history (including hypertension, diabetes mellitus) were collected using a self-reported questionnaire at baseline. Physical activity was classified as inactive (none), moderately active (<80 min per week), and very active (≥4 times per week and ≥20 min at a time). Blood pressure (BP) was measured by trained nurses at baseline, and the average of two readings was recorded. Weight and height were measured and body mass index (BMI) was calculated as weight in kilograms divided by the square of height in meters. For biochemical indexes, fasting blood glucose (FBG) was measured using the hexokinase/glucose-6-phosphate dehydrogenase method; triglyceride (TG) was measured using the GPO method; low-density lipoprotein cholesterol (LDL-C) and high-density lipoprotein cholesterol (HDL-C) were measured using a direct test method.

### Outcome: Stroke Incidence

The primary outcome of the study was newly diagnosed stroke (fatal or non-fatal), and the subtypes of stroke. Participants were followed from the index date, to the date of stroke or death, or until December 31, 2017, whichever came first. The follow-ups were performed by trained physicians who were blinded to the baseline data. For the participants without face-to-face follow-ups, outcome information was directly obtained by checking discharge summaries, medical records, and death certificates from provincial vital statistics offices. The International Classification of Diseases-10th Revision (ICD-10) codes were used to define stroke subtypes, which were categorized into IS (163), ICH (161–162), and SAH (160). Computed tomography and/or magnetic resonance imaging were routinely used for stroke diagnosis, and the diagnostic criteria were consistent across all participating hospitals. All stroke outcomes were validated by the Data Safety Monitoring Board and the Arbitration Committee for Clinical Outcomes.

### Statistical Analysis

Continuous variables were presented as median (interquartile ranges, IQR), and categorical variables were expressed as count (percentage). The group differences of continuous and categorical variables were compared by ANOVA and chi-squared tests, respectively. For each participant, person-years of follow-up were calculated from the index date until whichever stroke, death, or the last interview in 2016–2017 came first. Kaplan-Meier curves were generated and the log-rank test was employed to perform comparisons between the tertiles of TC change. Cox proportional hazards regression analysis was used to evaluate the risk of stroke and stroke subtypes, expressed as the hazard ratios (HRs) and 95% confidence intervals (CIs). Multivariate regression models were applied: model 1 was adjusted for age, sex, and TC at baseline; model 2 was adjusted for variates in model 1 plus smoking status, drinking status, physical activity, hypertension, diabetes mellitus, and BMI; model 3 was further adjusted for systolic BP and FBG in 2006. Meanwhile, HRs of each outcome were calculated for one standard deviation (SD) increase in TC change. Given the small sample size (*n* = 93) and different pathogenesis, SAH was not analyzed in our study. As 11 hospitals participated, models were used with a sandwich covariance matrix as a random effect to account for the potential confounding effect of different hospitals. Additionally, subgroup analyses stratified by age and other potential indicators were performed. All statistical analyses were performed using SAS, version 9.4 (SAS Institute Inc., Cary, NC, USA), and a two-sided value of *p* < 0.05 was considered statistically significant.

## Results

Out of the 70,999 enrolled participants (55,399 males and 15,600 females), 2,815 cases of stroke events were identified with a median follow-up period of 9.0 years. Among them, 3.5% (2,491/70,999) had IS, 0.5% (389/70,999) had ICH, and 0.1% (93/70,999) had SAH.

### Baseline Characteristics

The mean value of 2-year TC change in the three categories was −0.77, 0.00, and 0.88 mmol/L. There were significant differences in age, sex, smoking status, drinking status, physical activity, medical history, BP, BMI, and biochemical indexes among the tertiles of TC change (*p* < 0.001, Table 1). Those with the highest tertile of TC change were more likely to be younger, a current smoker or alcoholic, getting less exercise, or having lower baseline TC level.

**Table 1:** Baseline characteristics of participants according to the tertile of 2-year TC change.

**Variables**	**Total**	**TC change**			***p*** **-value**
		**T1**	**T2**	**T3**	
TC change, mmol/L	0.00 (−0.52, 0.58)	−0.77 (−1.14, −0.53)	0.00 (−0.16, 0.17)	0.88 (0.58, 1.38)	
Age, years	50.6 (42.7, 57.3)	50.8 (43.1, 57.4)	50.7 (42.7, 57.7)	50.3 (42.1, 56.9)	<0.001
Male, *n* (%)	55,399 (78.0)	18,400 (78.0)	18,275 (77.1)	18,724 (79.0)	<0.001
Current smoker, *n* (%)	24,633 (35.7)	8,221 (35.6)	7,811 (33.9)	8,601 (37.5)	<0.001
Current alcoholic, *n* (%)	27,834 (40.3)	9,245 (40.0)	8,747 (38.0)	9,842 (42.9)	<0.001
**Physical activity**					<0.001
Inactive, *n* (%)	6,446 (9.4)	2,017 (8.8)	2,028 (8.9)	2,401 (10.6)	
Moderately active, *n* (%)	51,202 (74.9)	17,220 (75.1)	17,226 (75.6)	16,760 (74.1)	
Very active, *n* (%)	10,701 (15.7)	3,692 (16.1)	3,537 (15.5)	3,472 (15.3)	
Hypertension, *n* (%)	28,847 (40.6)	9,706 (41.1)	9,307 (39.3)	9,834 (41.5)	<0.001
Diabetes mellitus, *n* (%)	5,922 (8.3)	2,216 (9.4)	1,743 (7.4)	1963 (8.3)	<0.001
SBP, mmHg	128.3 (116.7, 140.0)	129.3 (118.0, 140.0)	126.0 (115.0, 140.0)	128.7 (116.7, 140.0)	<0.001
DBP, mmHg	80.0 (77.3, 90.0)	80.0 (78.3, 90.0)	80.0 (76.7, 90.0)	80.0 (78.0, 90.0)	<0.001
BMI, kg/m^2^	24.9 (22.6, 27.2)	25.0 (22.7, 27.3)	24.7 (22.5, 27.1)	24.9 (22.8, 27.3)	<0.001
FBG, mmol/L	5.1 (4.7, 5.7)	5.2 (4.7, 5.8)	5.1 (4.6, 5.6)	5.1 (4.6, 5.7)	<0.001
TC, mmol/L	4.9 (4.3, 5.6)	5.4 (4.9, 6.1)	4.9 (4.3, 5.4)	4.4 (3.9, 5.1)	<0.001
TG, mmol/L	1.3 (0.9, 1.9)	1.3 (0.9, 2.0)	1.2 (0.8, 1.8)	1.3 (0.9, 2.0)	<0.001
LDL-C, mmol/L	2.3 (1.8, 2.9)	2.4 (1.9, 3.0)	2.3 (1.7, 2.8)	2.3 (1.8, 2.8)	<0.001
HDL-C, mmol/L	1.5 (1.3, 1.8)	1.5 (1.3, 1.8)	1.5 (1.3, 1.8)	1.5 (1.2, 1.7)	<0.001

*Values are number (percentage) for categorical variables and median (interquartile range) for continuous variables; TC, total cholesterol; SBP, systolic blood pressure; DBP, diastolic blood pressure; BMI, body mass index; FBG, fasting blood glucose; TG, triglyceride; LDL-C, low-density lipoprotein cholesterol; HDL-C, high-density lipoprotein cholesterol*.

### Correlation Between TC Change and Stroke Risk

**Figure 2:**
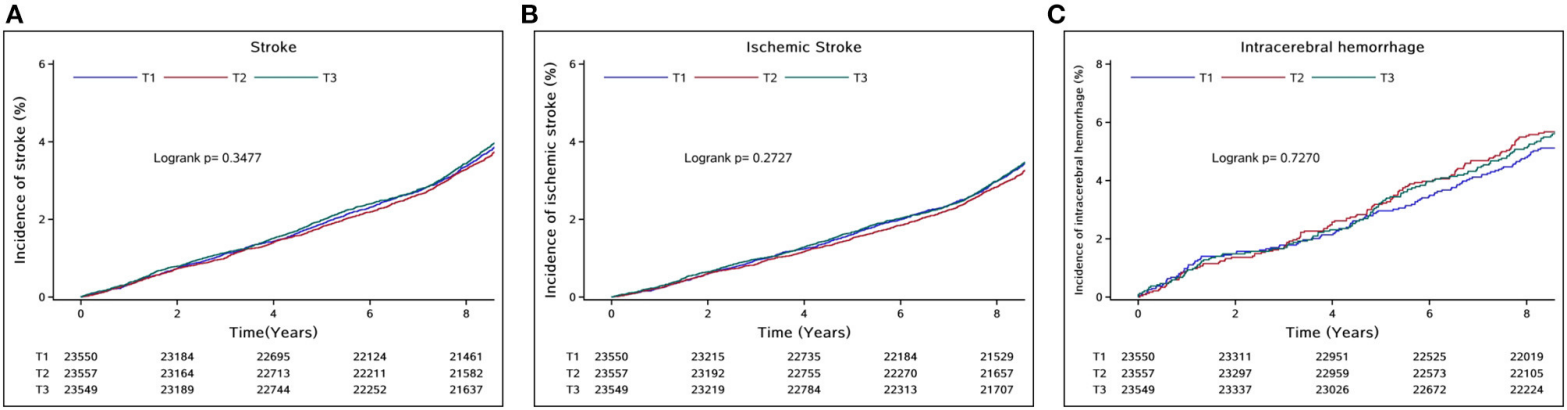
Cumulative incidences of **(A)** total stroke, **(B)** ischemic stroke, and **(C)** intracerebral hemorrhage according to the tertile of 2-year TC change.

The incidence rate of all-type stroke per 1000 person-years from the lowest to highest tertile of TC change was 4.79, 4.42, and 4.77, respectively. In univariate analysis, the cumulative incidences of total stroke, IS, and ICH were not statistically different among the three groups (log-rank test, all *p* > 0.05, Figure 2). After adjusting for baseline TC levels and potential confounding factors, the association between TC change and total stroke became significant in the highest tertile. In model 3, the adjusted HR values were 1.00 (reference), 1.01 (0.92–1.11), and 1.17 (1.06–1.29) from the lowest to the highest tertile of TC change. Similar results were obtained in participants with incident IS, as those in the highest tertile had an 18% relative risk increase (HR 1.18, 95% CI 1.06–1.36). Significantly, a 1.07-fold and 1.08-fold increased risk of total stroke and IS were observed per SD (1.04 mmol/L) increase in TC change. However, no association was found between ICH and TC change, additional information is given in Table 2.

**Table 2:** Hazard ratios (HR) for stroke according to the tertile of 2-year TC change.

	**T1**	**T2**	**T3**	***p*** **for trend**	**Per 1 SD increase^†^**
**Total stroke**					
Events, *n* (%)	959 (4.1)	892 (3.8)	964 (4.1)		
Incidence rate, per 1,000 person-years	4.79	4.42	4.77		
Model 1	Ref.	1.02 (0.93–1.12)	1.22 (1.10–1.34)	<0.001	1.10 (1.06–1.15)
Model 2	Ref.	1.00 (0.91–1.10)	1.17 (1.06–1.30)	0.002	1.08 (1.03–1.13)
Model 3	Ref.	1.01 (0.92–1.11)	1.17 (1.06–1.29)	0.002	1.07 (1.02–1.12)
**Ischemic stroke**					
Events, *n* (%)	859 (3.6)	782 (3.3)	850 (3.6)		
Incidence rate, per 1,000 person-years	4.28	3.87	4.2		
Model 1	Ref.	1.01 (0.91–1.12)	1.22 (1.10–1.36)	<0.001	1.11 (1.06–1.17)
Model 2	Ref.	0.99 (0.89–1.10)	1.18 (1.06–1.31)	0.003	1.09 (1.03–1.14)
Model 3	Ref.	0.99 (0.90–1.10)	1.18 (1.06–1.31)	0.003	1.08 (1.03–1.13)
**Intracerebral hemorrhage**					
Events, *n* (%)	123 (0.5)	133 (0.6)	131 (0.6)		
Incidence rate, per 1,000 person-years	0.61	0.65	0.64		
Model 1	Ref.	1.11 (0.86–1.42)	1.13 (0.86–1.48)	0.379	1.03 (0.91–1.16)
Model 2	Ref.	1.12 (0.86–1.45)	1.07 (0.90–1.42)	0.663	1.00 (0.88–1.13)
Model 3	Ref.	1.12 (0.86–1.46)	1.07 (0.80–1.42)	0.659	0.99 (0.88–1.13)

*Model 1: adjusted for age, sex, and total cholesterol (TC) at baseline*.

*Model 2: adjusted for variables in model 1, hypertension, diabetes mellitus, smoking status, drinking status, physical activity, and body mass index*.

*Model 3: adjusted for variables in model 2 plus systolic blood pressure and fasting blood glucose*.

† *1 SD increase in 2-year TC change was equal to 1.04 mmol/L*.

### Subgroup Analysis for Incidence of Stroke Subtypes

Age, sex, hypertension, and diabetes mellitus were stratified for further subgroup analyses of the association between TC change and the incidence of stroke subtypes, both IS and ICH. No significant association was found in each subgroup (Table 3).

**Table 3:** Multivariate-adjusted hazard ratios (HR)^†^ for stroke subtypes according to the tertile of 2-year TC change, stratified by sex and selected risk factors.

	**T1**	**T2**	**T3**	***p*** **for interaction**
**Ischemic stroke**					
Age				0.425
<60	Ref.	0.96 (0.84–1.10)	1.11 (0.97–1.28)	
≥60	Ref.	1.04 (0.89–1.22)	1.24 (1.05–1.46)	
Sex				0.422
Women	Ref.	1.12 (0.82–1.53)	1.10 (0.78–1.55	
Men	Ref.	0.98 (0.88–1.09)	1.19 (1.06–1.33)	
Hypertension				0.574
No	Ref.	0.97 (0.82–1.16)	1.24 (1.04–1.49	
Yes	Ref.	1.00 (0.88–1.14)	1.14 (1.00–1.30)	
Diabetes mellitus				0.799
No	Ref.	0.98 (0.88–1.10)	1.17 (1.04–1.31)	
Yes	Ref.	1.06 (0.83–1.36)	1.23 (0.96–1.58)	
**Intracerebral hemorrhage**					
Age				0.228
<60	Ref.	0.97 (0.70–1.36)	0.97 (0.68–1.38)	
≥60	Ref.	1.41 (0.91–2.18)	1.22 (0.75–1.97)	
Sex				0.93
Women	Ref.	1.46 (0.74–2.90)	1.40 (0.66–2.97)	
Men	Ref.	1.07 (0.81–1.42)	1.02 (0.75–1.39)	
Hypertension				0.98
No	Ref.	1.11 (0.69–1.79)	1.15 (0.69–1.93)	
Yes	Ref.	1.12 (0.82–1.54)	1.03 (0.73–1.44)	
Diabetes mellitus				0.273
No	Ref.	1.01 (0.76–1.34)	0.96 (0.71–1.31)	
Yes	Ref.	2.17 (1.07–4.41)	1.97 (0.92–4.20)	

† *Adjusted for age, sex, hypertension, diabetes mellitus, smoking status, drinking status, physical activity, body mass index, systolic blood pressure, fasting blood glucose, and total cholesterol at baseline*.

## Discussion

In this community-based, large-scale, and long-term follow-up study, we found that the 2-year change in TC level was an independent predictor for total stroke and IS, but not for ICH. Our study demonstrated that per 1.04 mmol/L increment in TC change yielded a 7% increased risk of incident total stroke and 8% of incident IS during a median follow-up of 9.0 years.

It was noteworthy that the mean age of participants from the lowest to the highest tertiles of TC change reached statistical significance (*p* < 0.001), while the difference in age value was minor. The confounding factor, age, was adjusted in all the three multivariate models. Moreover, subgroup analysis stratified by age (<60 vs. ≥60) was conducted to minimize the age-related stroke risk attributed to lipid deposition. That is to say, the possibility of selection bias regarding age was small.

One previous study reported that increased TC was associated with an elevated risk of ischemic heart disease among young adults when compared to subjects with sustained TC levels (adjusted HR 1.09, 95% CI 1.06–1.11) (13). Our study adds to the evidence that a higher level of TC change was associated with an increased risk of overall stroke in the general population, particularly in patients with IS rather than ICH. Noteworthy was the downward tendency of baseline TC value from the lowest to the highest tertile of TC change; we assumed that participants with a higher baseline TC level would pay more attention to lipid-lowering strategy, while those with a lower baseline TC level would be over-optimistic toward lipid trend and thus ignore the dynamic monitoring of lipid level. As the abovementioned study used a time frame of 2 years and the participants in Kailuan study were followed up biennially, we thus chose a 2-year interval. Compared with single-point measurement of cholesterol endorsed by major guidelines (16, 17), visit-to-visit cholesterol monitor has gradually attracted concern. Besides the change of cholesterol defined by two measurements, variation of cholesterol defined by at least three measurements was associated with adverse cardiovascular events as well (18–21). Atherosclerosis is a progressive process; the increase in lipid level could accelerate lipid deposition and plaque formation in comparison to those with declined or sustained level. Our results support the view that repeated measurements of TC would help to identify individuals at higher risk of stroke and further highlight the importance of cholesterol monitoring in stroke prevention.

No correlation was found between TC change and incident ICH in our study, whereas a meta-analysis of 23 prospective studies including a total of 1,430,141 participants reported that per 1 mmol/L increase in TC yielded a 15% decreased risk of hemorrhagic stroke (95% CI 0.80–0.91) (22). Additionally, some studies demonstrated that lower TC level was correlated with increased hemorrhagic risk and adverse clinical outcomes in ICH patients (11, 23, 24). The underlying mechanisms may be attributed to the destruction of endothelial integrity, necrosis of medial smooth muscle cells, and the reduction of platelet aggregability associated with lower lipid levels (25–27). It is the significantly lower lipid concentration that matters, not its dynamic change. When it comes to our study, the baseline TC level was within the normal physiological range (28). It would be of great clinical significance to further investigate the risk threshold of TC on hemorrhagic stroke, especially for subjects with an indication for lipid-lowering therapy.

Our study used the visit-to-visit TC change instead of single-point TC value to make risk assessments for incident total stroke and stroke subtype in the general population. Nonetheless, there are still several limitations. First, there may still be some unmeasured environmental and social confounding factors embedded in this observational cohort, even though we have excluded subjects with previous lipid-lowering therapy and adjusted for a series of vascular risk factors. Secondly, because of the lack of vascular evaluation and structural imaging information, we cannot classify the subtypes of ischemic stroke and explore the associations a step forward. Thirdly, we only focused on 2-year TC change in the present study; other atherogenic lipid parameters with repeated measurements merit further investigation. Moreover, homogeneous Chinese participants living in a similar environment may limit the generalizability of the results.

In conclusion, increased 2-year TC change is associated with an elevated risk of incident total stroke and ischemic stroke, irrespective of the baseline TC value. Maintaining a sustained ideal level of TC may reduce the incidence of future cerebrovascular ischemic events.

## Data Availability Statement

The raw data supporting the conclusions of this article will be made available by the authors, without undue reservation.

## Ethics Statement

The study was conducted in accordance with the Declaration of Helsinki and was approved by the Ethics Committees of the Kailuan General Hospital and Beijing Tiantan Hospital. The patients/participants provided their written informed consent to participate in this study.

## Author Contributions

YW performed the experiments, interpreted the results of statistical analysis, and drafted the manuscript. AW and YZ performed data analysis. SW commented on the drafts. XZ revised the manuscript for intellectual content. All authors read and approved the final manuscript and agreed to be accountable for all aspects of the work.

## Funding

The Kailuan study was supported by grants from the Chinese Academy of Medical Sciences Innovation Fund for Medical Sciences (2019-I2M-5-029), Beijing Natural Science Foundation (Z200016), and Beijing Municipal Committee of Science and Technology (Z201100005620010).

## Conflict of Interest

The authors declare that the research was conducted in the absence of any commercial or financial relationships that could be construed as a potential conflict of interest.

## Publisher's Note

All claims expressed in this article are solely those of the authors and do not necessarily represent those of their affiliated organizations, or those of the publisher, the editors and the reviewers. Any product that may be evaluated in this article, or claim that may be made by its manufacturer, is not guaranteed or endorsed by the publisher.
