# Supplementary material for: Two-Year Change in Serum Total Cholesterol Is Associated With Incident Ischemic Stroke: Results From the Kailuan Study
Source: Front Neurol. 2021 Sep 29;12:710083. doi: 10.3389/fneur.2021.710083 (PMC8511704; doi:10.3389/fneur.2021.710083)
Supplement: Supplementary file 1 [file Table_1.DOCX]

Supplementary Table 1. Characteristics of included and excluded participants.

| Variables | Total | Included | Excluded | *P-*value |
| --- | --- | --- | --- | --- |
| n (%) | 101510 | 70999 (69.9) | 30511 (30.1) |  |
| Age, years | 52.0 (43.7, 59.4) | 50.6 (42.7, 57.3) | 55.6 (48.8-64.7) | <0.001 |
| Male, n (%) | 81110 (79.9) | 55399 (78.0) | 25711 (84.3) | <0.001 |
| BMI, kg/m^2^ | 24.9 (22.6, 27.2) | 24.9 (22.6, 27.2) | 24.8 (22.6, 27.2) | 0.776 |
| SBP, mmHg | 130.0 (119.3, 141.3) | 128.3 (116.7, 140.0) | 130.0 (120.0, 150.0) | <0.001 |
| DBP, mmHg | 80.0 (78.7, 90.0) | 80.0 (77.3, 90.0) | 81.0 (79.3, 90.0) | <0.001 |
| FBG, mmol/L | 5.1 (4.7, 5.7) | 5.1 (4.7, 5.7) | 5.1 (4.7, 5.8) | <0.001 |
| Current smoker, n (%) | 33795 (33.3) | 24633 (35.7) | 9162 (31.4) | <0.001 |
| Current alcoholic, n (%) | 36652 (36.1) | 27834 (40.3) | 8818 (30.2) | <0.001 |
| Hypertension, n (%) | 13004 (12.8) | 7701 (10.9) | 5303 (17.4) | <0.001 |
| Diabetes mellitus, n (%) | 3250 (3.2) | 1840 (2.6) | 1410 (4.6) | <0.001 |
| Physical activity |  |  |  | <0.001 |
| Inactive, n (%) | 8499 (8.4) | 6446 (9.4) | 2053 (7.1) |  |
| Moderately active, n (%) | 73541 (72.5) | 51206 (74.9) | 22335 (77.1) |  |
| Very active, n (%) | 15281 (15.1) | 10701 (15.7) | 4580 (15.8) |  |
| TC, mmol/L | 5.1 (4.7, 5.7) | 4.9 (4.3, 5.6) | 5.1 (4.7, 5.6) | 0.177 |
| TG, mmol/L | 1.3 (0.9, 1.9) | 1.3 (0.9, 1.9) | 1.3 (0.9, 2.0) | <0.001 |
| LDL-C, mmol/L | 2.3 (1.8, 2.8) | 2.3 (1.8, 2.9) | 2.3 (1.8, 2.8) | 0.002 |
| HDL-C, mmol/L | 1.5 (1.3, 1.8) | 1.5 (1.3, 1.8) | 1.5 (1.3, 1.8) | <0.001 |

Values are n (%) for categorical variables and median (IQR) for continuous variables; BMI, body mass index; SBP, systolic blood pressure; DBP, diastolic blood pressure; FBG, fasting blood glucose; TC, total cholesterol; TG, triglyceride; LDL-C, low-density lipoprotein cholesterol; and HDL-C, high-density lipoprotein cholesterol.
